# Nephron sparing surgery: A single institution experience

**DOI:** 10.4103/0970-1591.30260

**Published:** 2007

**Authors:** S. Agrawal, M. S. Jha, N. Khurana, M. S. A. Ansari, D. Dubey, A. Srivastava, R. Kapoor, A. Kumar, M. Jain, A. Mandhani

**Affiliations:** Department of Urology and Renal Transplantation, Sanjay Gandhi Post Graduate Institute of Medical Science, Lucknow, (UP), India; * Department of Pathology, Sanjay Gandhi Post Graduate Institute of Medical Science, Lucknow, (UP), India

**Keywords:** Kidney, kidney diseases, nephron sparing surgery

## Abstract

**Objective:**

To report our experience in managing various benign and malignant renal tumors with nephron-sparing surgery.

**Materials and Methods:**

Records of patients who underwent nephron-sparing surgery (NSS) either through open or laparoscopic approach between May 1997 and June 2006 at our institution were reviewed. Patient and tumor-related characteristics, treatment modality and complications were noted.

**Results:**

There were 26 patients (29 renal units), including three with bilateral lesions who underwent nephron-sparing surgery. Mean age at surgery was 47.0 years (range 16-67 years). Mean tumor size was 4.7 cm (range 2-7.5 cm). Mean warm ischemia time was 41 min and 32.5 min, operative time 158 min and 186 min and blood loss 200ml and 85 ml in open (n=24) and laparoscopic approach (n=2) respectively. Complications were seen in five (19.2%) patients of whom two had postoperative bleeding requiring nephrectomy in one and angioembolization in another. One patient with persistent urinary leak required intervention. Local wound infection in one patient and incisional hernia in another were surgically managed. Histopathological profile revealed 13 (44.8%) benign lesions which included angiomyolipoma (eight), simple cyst (two), cortical adenoma (one), metanephric adenoma (one) and myelolipoma (one). The remaining 16 (55.2%) malignant lesions included renal cell carcinoma (15) and metastatic adenocarcinoma (one). At a mean follow-up of 38.6 months (range 1-91) no patient had local recurrence or distant metastasis. Cancer-specific survival was 100% and overall survival was 92.3%.

**Conclusions:**

Nephron-sparing surgery is a safe and effective alternative to nephrectomy in both benign and malignant lesions of the kidney.

Enthusiasm for nephron-sparing surgery (NSS) has been stimulated by several trends, including advances in renal imaging, improved surgical techniques and methods to prevent ischemic renal injury, better postoperative management, such as renal replacement therapy and long-term prospective cancer-free survival data.[1] Nephron-sparing surgery may be performed safely and cost-effectively with low morbidity, preservation of renal function, a low local recurrence rate and high patient satisfaction.[2,3] In India, the rate of incidentally detected renal tumors is much lower that is 8% as compared to 50% in western countries because most of these cases present at an advanced stage.[4] Lack of regular health checkup as well as late consultation are the two main causes for not getting suitable cases for NSS. A survey of the existing literature revealed limited experience with NSS in India. Herein, we analyze our experience with nephron-sparing surgery with regards to the demographic profile of cases, clinical presentation, surgical and adjuvant therapy, complications and overall survival.

## MATERIALS AND METHODS

**Figure 1:**
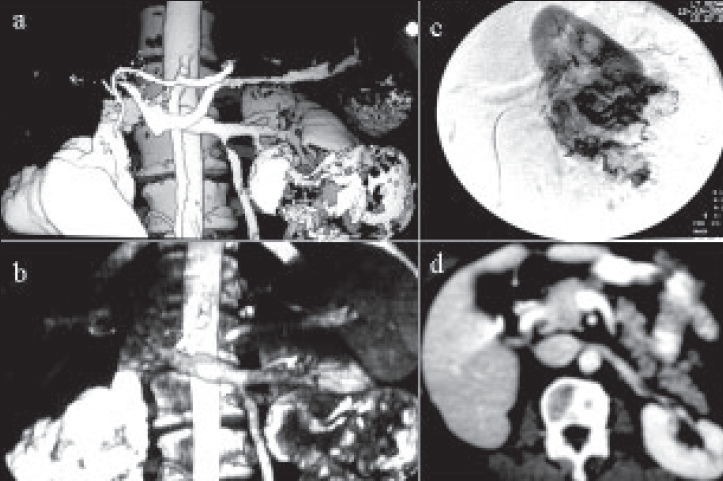
Spiral CT angiogram showing bilateral renal tumor. Right side radical nephrectomy and left lower polar partial nephrectomy was performed. a) and b) 3-D view, c) renal DSA of left side, d) postoperative follow-up CT scan after 3.8 years

Records of patients who had undergone NSS for various benign and malignant lesions between May 1997 and June 2006 were reviewed. Preoperative evaluation comprised urinalysis, chest radiograph, liver and renal function tests and abdominal CT. Magnetic resonance imaging (MRI), digital subtraction angiography [Figure 1], color Doppler scan and radionuclide imaging were obtained wherever indicated.

### Operative technique

*Open approach*: An extraperitoneal flank approach through the 11^th^ or 12^th^ rib bed was preferred. Extra-gerotal dissection was performed. Renal artery and vein were mobilized separately. After clamping renal artery, ice slush was placed around the kidney for 15 min to obtain regional hypothermia. Gerotas fascia and renal capsule were sharply incised around the lesion leaving a 0.5 to 1.0 cm margin of normal appearing parenchyma beyond the visual limits of the tumor. Frozen section of the tumor bed was obtained in cases of suspected RCC in the initial eight cases and later on this practice was abandoned due to a low yield. Segmental renal vessels were individually ligated with 5-O' prolene. Disruptions in the collecting system were detected by instilling dilute solution of methylene blue through a preplaced ureteric catheter and oversewn with 4-O' vicryl or polydioxanone suture. Surgicel and gelfoam spongstran bolster were then placed on the cut surface and 2-O' vicryl sutures were used to approximate the remaining renal parenchyma. A double J stent was placed in cases where the pelvicalyceal system was opened.

**Figure 2:**
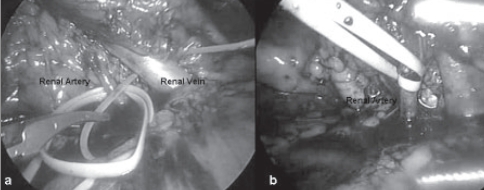
a) and b) Vascular tapes applied around renal vessels for vascular control during laparoscopic approach

*Laparoscopic approach*: For laparoscopic partial nephrectomy 4-port technique was used. Hilar control was obtained with the help of vascular loops (SURG - I - LOOP; SCANLAN International, Netherlands) placed around the artery and vein. These loops were brought out extracorporally through small incisions and traction was applied to occlude the vessels [Figure 2]. No regional hypothermia was attained. Patients were followed up with serum creatinine, urinalysis and renal sonography or computerized tomography scan.

## RESULTS

Table 1 lists the demographic and operative data of the patients' cohort. There were 26 patients (29 renal units), including three with bilateral lesions who underwent nephron-sparing surgery. Of 26 patients, 24 underwent NSS by open technique and two by laparoscopic technique. Mean age at surgery was 47.0 years (range 16-67 years). Mean tumor size was 4.7 cm (range 2-7.5 cm). Of the 26 patients, 18(69.2%) had symptomatic lesions and the remaining eight (30.8%) were incidentally detected. Partial nephrectomy was performed as an elective procedure in 22 (84.6%) cases while one case had a relative indication for NSS – renal tumor with contralateral pelvi-ureteric junction obstruction with mild impaired renal function. Three patients with bilateral synchronous renal tumors warranted a nephron-sparing approach on each side. Nephron-sparing surgery was performed as a staged procedure (with a minimum duration of one month) with the less involved side done first to obviate the need for temporary dialysis if acute tubular necrosis developed after NSS.

**Table 1:** Demographic and operative data n = 26 patients

Mean age (years)	46.0
No. sex:	
Male	13
Female	13
No. side:	
Rt	16
Lt	7
Bl*	3
Presentation	
Symptomatic	18
Incidental	8
Mean tumor size (cm)	4.7
Angiomyolipoma	6.0
Renal cell carcinoma	3.8
Others	4.5
Mean estimated blood loss (ml)	200
Mean warm ischemia time (min)	41
Mean operative time (min)	158
Mean S. Creatinine (mg/dl)	
Preop	1.13
Postop	1.27
Mean hospital stay (days)	8

* 3 cases were bilateral renal masses, 2 male and 1 female Publications

Table 2 reveals the histopathological profile of 29 surgically removed specimens. There were 13 (44.8%) benign lesions which included eight angiomyolipomas. Two young patients with complex, polar, Bosniak Type III cysts, with calcifications and irregular margins were operated upon for suspected malignancy, but they revealed to be simple cysts in each. The remaining 16 (55.2%) specimens were compatible with malignant lesions, in which renal cell carcinoma (RCC) accounted for 15 cases. Clear cell carcinoma was the commonest histological cell type. There was one case of metastatic adeno-carcinoma whose origin could not be detected as the case was lost to follow-up.

**Table 2:** Histopathological characteristics of 29 partial nephrectomy specimens

**Benign pathology**	
Angiomyolipoma	8
Cyst	2
Renal cortical adenoma	1
Metanephric adenoma	1
Myelolipoma	1
Total	13
**Malignant tumors**	
Renal cell carcinoma	
Clear cell	11
Papillary	4
Metastatic adeno-ca	1
Total	16

The majority – 11 (73.3%) of the RCC were less than 4 cm in size. Seven of RCC were located at the upper pole, four at lower pole, while four were found to be in central position. Pathological tumor staging was determined according to the 1997 TNM classification.[5] All the 15(100%) cases of RCC were of low stage (T1) and 13 (86.7%) had a favorable Fuhrmann's grade (GD II or less). None of the specimens revealed positive surgical margins or evidence of vascular invasion.

**Figure 3:**
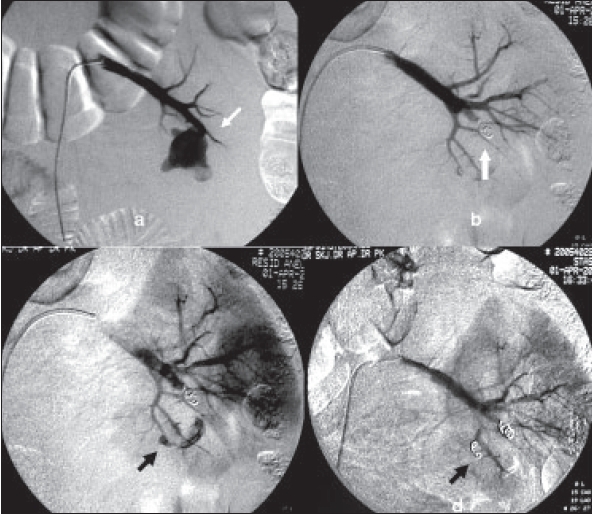
a) Angiography showing pseudoaneurysm arising from the lobar artery (white arrow) b) feeding vessel being plugged by aneurysm coil (white arrow) c) and d) another pseudoaneurysm being plugged by aneurysm coil (black arrow)

Minor to major complications were seen in five (19.2%) patients. Two of the cases had postoperative bleeding. One was reactionary bleeding for which re-exploration was done and nephrectomy performed in view of persistent intraoperative hemodynamic instability. The other case had hemorrhage from pseudo-aneurysm arising from the lobar artery, which was managed by angioembolization [Figure 3]. One case had prolonged drain output (urine) and was diagnosed to have urinary leak from the transected lower pole in which the double J stent had migrated down the ureter and thus was not draining the pelvicalyceal system adequately. It was managed by placement of a nephrostomy tube. Local wound infection in one case was successfully managed by wound toileting and subsequent secondary suturing. Incisional hernia in one patient presenting after 21/2 years was successfully repaired by mesh hernioplasty.

Mean warm ischemia time, operative time and blood loss was 41 min, 158 min and 200 ml in the open approach and 32.5 min, 186 min and 85 ml in the laparoscopic approach respectively. Mean preoperative serum creatinine was 1.13 mg/dl (range 0.7-1.5 mg/dl). Mean serum creatinine at third postoperative month was 1.27 mg/dl (range 0.6-2.0 mg/dl). Two patients with bilateral renal masses with normal renal function developed chronic renal insufficiency (creatinine ≥ 1.5) postoperatively. They were managed conservatively and are under regular follow-up.

Follow-up data was available in 25 (96.2%) cases. At a mean follow-up of 38.6 months (range 1-91), none of the patients had local recurrence or distant metastasis. One patient who had a prior history of gallstone pancreatitis with diabetes and hypertension died four months later of hepatic encephalopathy. Another patient with bilateral large renal tumors, who for the purpose of renal preservation, underwent simultaneous radical nephrectomy on one side and bench partial nephrectomy for complex, lower and mid-polar tumor invading into pelvicalyceal system near hilum on the opposite side, subsequently died of acute tubular necrosis with acute respiratory distress syndrome and multi-organ failure. Overall survival was 92.3% and cancer-specific survival was 100%.

## DISCUSSION

Standard indications for NSS fall into three categories, i.e. absolute, relative and elective. Absolute indications include circumstances where radical nephrectomy would render the patient anephric, with a subsequent immediate need for dialysis. This includes patients with bilateral RCC or RCC involving a solitary functioning kidney. Three of our patients had bilateral renal tumors. Relative indications for NSS include patients with unilateral RCC and a functioning contralateral kidney when the contralateral kidney is affected by a condition that threatens its future function, e.g. calculus disease, chronic pyelonephritis. It also includes patients with hereditary forms of RCC such as von Hippel-Lindau disease (VHL). Elective indications for NSS include patients with localized unilateral RCC and a normal contralateral kidney.[6] The role of partial nephrectomy in angiomyolipoma and oncocytoma has already been established. [7]

Nephron-sparing surgery is a technically advanced procedure requiring considerable expertise. Intraoperative issues may be attributable to time limitations caused by warm ischemia, wherein the tumor must be excised and precise hemostatic repair performed expeditiously, especially when considering laparoscopic approach. Our modest surgical experience regarding NSS compares favorably with the other series reported so far[8] regarding estimated blood loss, operative time, convalescence and hospital stay. After having accrued sufficient experience with the open approach, laparoscopic partial nephrectomy in two patients was successfully performed with good oncological (negative surgical margin) and functional outcome.

Although radical nephrectomy remains the standard treatment for localized RCC, different series have reported similar cancer-specific survival or progression-free interval at five or 10 years in patients who underwent radical nephrectomy or NSS for localized RCC.[9] In a recent study from the Memorial Sloan-Kettering Center, Herr *et al*[10] reported the 10-year results of elective NSS for patients with a normal contralateral kidney. With a mean tumor size of 3 cm and with predominantly low-grade, low-stage tumors, 69 of the 70 patients (98.5%) had no local recurrence and 68 (97%) survived free of metastasis. Thus our series comprise majority - 22/26 (84.6%) of NSS, performed as an elective procedure. Although the long-term functional advantage of NSS when there is a normal contralateral kidney remains to be definitively shown, the benefits might include a decreased risk of progression to chronic renal insufficiency and end-stage disease.[11] The oncological results presented in our series are encouraging. None of the specimens was found to have a positive margin on final histopathological analysis. Cancer-specific survival and overall survival at a mean follow-up of 38.6 months (range 1–91) was 100% and 92.3% respectively. Overall negative tumor margin status along with the relative majority of patients with low-grade 13 (86.7%) and a favorable tumor stage 15(100%), would suggest durable outcome even during longer follow-up. For tumors smaller than 4 cm, the local recurrence rate has been estimated between 1.5-4%.[1,12] Prior series assessed the prognostic significance of tumor stage, grade, size, laterality and margin status on the long-term outcomes of open NSS.[1,12] In the absence of cancer recurrence or cancer-related death as an end point, the present study could not analyze the factors affecting disease outcome. Similarly, given the lack of cancer mortality, estimates of actuarial five-year survival in this series are not warranted.

Prolonged acute tubular necrosis with or without clinically overt renal failure may occur in 6.3% (range 0.7-7.3%) of patients.[13] In the current series, two patients who developed chronic renal insufficiency had bilateral renal masses. Considering the relatively short cold ischemia time of 38 and 32 min respectively, it is unlikely that ischemic insult per se had a major role in the gradually worsening renal function in these patients. Excisional loss of functioning parenchyma is probably the cause for deteriorating renal function. Long-term efficacy of NSS in preserving renal function is evident by the fact that none of the patients in the present series required renal replacement therapy.

Despite reconstructing renal remnant over a hemostatic agent, such as oxidized cellulose (surgicel), bleeding is an important complication. Van Poppel *et al*[14] reported hemorrhage in 7.9% of 76 patients and suggested that larger tumor size and a central location correlated with the risk of postoperative hemorrhage. In the present series, two of 26 cases required re-exploration and underwent nephrectomy in one and angioembolization in the other.

The cumulative incidence of urinary fistula following partial nephrectomy is reported as 6.5%.[6] In the current series urinary leakage developed in only one case which resulted possibly due to down migration of the stent causing obstruction.

Review of the literature reveals that when NSS is performed with attention to excising a perimeter of grossly normal-appearing parenchyma, sending specimens for intraoperative frozen section may result in an unnecessary expense without providing meaningful and reliable information.[15] Hence, except in the initial few cases, no attempt was made to routinely obtain frozen section from the tumor bed.

We also recognize the shortcomings of our study – mainly the relative scarcity of numbers, inherent indolent nature of the favorable stage and low-grade renal tumors detected and the need for larger follow-up of 10 years to document definitive oncological outcome. However, our experience reaffirms the clinical relevance of nephron-sparing surgery with significant but manageable complications.

## CONCLUSION

Nephron-sparing surgery is a safe and effective alternative to nephrectomy in both benign and malignant lesions of the kidney. Overall functional and oncological outcomes are quite encouraging. Patients with NSS performed for bilateral renal masses should be followed closely for possible renal deterioration. Though technically feasible, it needs reasonable expertise to achieve the best functional and oncological outcome with minimal complications.
